# A New Means to Generate Liposomes by Rehydrating Engineered Lipid Nanoconstructs

**DOI:** 10.3390/mi16020138

**Published:** 2025-01-25

**Authors:** Yuqi Huang, Ziqian Xu, Umit Celik, Christopher F. Carnahan, Roland Faller, Atul N. Parikh, Gang-yu Liu

**Affiliations:** 1 Department of Chemistry, University of California, Davis, CA 95616, USA; yqhuang@ucdavis.edu (Y.H.); zqnxu@ucdavis.edu (Z.X.);; 2 Biophysics Graduate Group, University of California, Davis, CA 95616, USA; cfcarnahan@ucdavis.edu (C.F.C.); anparikh@ucdavis.edu (A.N.P.); 3 Department of Chemical Engineering, University of California, Davis, CA 95616, USA; 4 Department of Chemical Engineering, Texas Tech University, Lubbock, TX 79409, USA; roland.faller@ttu.edu; 5 Department of Biomedical Engineering, University of California, Davis, CA 95616, USA

**Keywords:** atomic force microscopy (AFM), lipid, 1-palmitoyl-2-oleoyl-sn-glycero3-phosphocholine (POPC), liposome, microfluidic, 3D nanoprinting

## Abstract

The concept and feasibility of producing liposomes by rehydrating engineered lipid nanoconstructs are demonstrated in this study. Nanoconstructs of 1-palmitoyl-2-oleoyl-sn-glycero-3-phosphocholine (POPC) were produced using a microfluidic delivery probe integrated with an atomic force microscope. The subsequent rehydration of these POPC constructs led to the formation of liposomes, most of which remained adhered to the surface. The size (e.g., diameter) of the liposomes could be tuned by varying the lateral dimension of the lipid constructs. Hierarchical liposomal structures, such as pentagons containing five liposomes at the corners, could also be designed and produced by depositing lipid constructs to designated locations on the surfaces, followed by rehydration. This new means allows for regulating liposomal sizes, distributions, and compositions. The outcomes benefit applications of liposomes as delivery vehicles, sensors, and building blocks in biomaterials design. The ability to produce hierarchical liposomal structures benefits numerous applications such as proto-cell development, multiplexed bio-composite materials, and the engineering of local bio-environments.

## 1. Introduction

Liposomes are vesicular structures mainly consisting of lipid bilayers [1]. As biomembrane-based analogous, liposomes are highly biocompatible and have been used for various applications including drug delivery [2,3,4,5], biomimetics [6,7], and cosmetics [8], as well as applications in the food and agriculture industries [9,10,11]. In addition, liposomes provide a unique local environment for scientific research, as they provide enclosures for engineered proto-cells [12,13]. Commonly used methods of preparing liposomes include lipid thin-film rehydration [14,15,16,17], emulsion-based methods [18,19,20,21,22,23,24,25,26,27,28,29,30,31], and microfluidic-based technologies [32,33,34,35,36]. The emulsion-based methods are simple and have high throughput but often face the limitations of broad size distribution in conjunction with the challenges of low stability or short shelf life. Thin-film rehydration allows for longer shelf life, as thin films are more stable than liposomes in emulsion, which also has the advantage of simplicity. However, this method also has limitations of broad size distribution among the resulting liposomes, thus requiring separation to attain the desired sizes. Often, these methods involve the use of organic solvents during production; thus, biocompatibility and sustainability are in question. The microfluidics method provides a means of forming liposomes with much narrower size distributions but is challenged by the need to use the liposomes immediately after production, as structural integrity becomes an issue during transportation and long-term storage. More advanced approaches include liposome formation via the hydration of electrospray-deposited lipid molecules, which enables a higher degree of control over the size of the initial lipid construct than the commonly used method [37]. The consistent production of ultrasmall lipid constructs and hierarchical lipid construction by design still remains challenging and is essential in order to realize modern applications such as tissue and biodevice engineering.

This work reports a new methodology to fill these voids. The new approach consists of two key steps: the 3D nanoprinting of lipids or lipid composites into designed sizes and geometry [38], followed by rehydrating these constructs to form liposomes. The lipid constructs are relatively dry, thus exhibiting high stability during transportation and storage. The size of liposomes, as will be shown in this report, is directly correlated with the dimension and geometry of the lipid constructs. Thus, by tuning the sizes of the lipid constructs, we can attain liposomes with designed sizes. Taking advantage of the positioning accuracy of this methodology, this work will also demonstrate the feasibility of producing designed hierarchical structures of liposomes. The new means of producing liposomes enables the achievement of designed sizes and compositions, which shall advance the applications of drug delivery and liposome-based biomaterial engineering. The ability to produce hierarchical liposomal structures also paves the way for numerous applications such as proto-cell development, multiplexed bio-composite materials, and engineered local bio-environments.

## 2. Experimental Section

### 2.1. Materials and Supplies

Glass slides and glass coverslips were purchased from Fisher Scientific (Pittsburgh, PA, USA). Reagents were used without further purification. Glycerol (>99%), sucrose (99.5%), and chloroform (99.8%) were purchased from Sigma–Aldrich (St. Louis, MO, USA). DPBS was purchased from Fisher Scientific (Pittsburgh, PA, USA). Ethanol (200-proof pure ethanol) was purchased from Koptec (King of Prussia, PA, USA). Milli-Q water (MQ water, 18.2 MΩ·cm at 25 °C) was produced by a Milli-Q water purification system (EMD Millipore, Billerica, MA, USA). Nitrogen gas (99.999%) was purchased from Praxair, Inc. (Danbury, CT, USA, King of Prussia, PA, USA). The 1-palmitoyl-2-oleoyl-sn-glycero-3-phosphocholine (POPC) and 1,2-dioleoyl-sn-glycero-3-phosphoethanolamine-N-(7-nitro-2-1,3-benzoxadiazol-4-yl) (NBD-PE) were purchased from Avanti Lipids, Inc. (Alabaster, AL, USA).

### 2.2. Preparation of Glass Supports

Coverslips were first cleaned with copious amounts of water, followed by ethanol. After cleaning, the glass coverslips were dried under nitrogen flow, followed by plasma-cleaning using air plasma at RF power = 18 W for 5 min with a plasma cleaner (PDC-32G, Harrick Plasma, Ithaca, NY, USA).

### 2.3. Contact Angle Measurements

Contact angles were measured using a VCA Optima Contact Angle Measurement system (AST Products, Billerica, MA, USA), following well-established protocols [39,40,41,42,43]. A total of 3 µL of the designated liquid was dispensed on the surface using a high-performance liquid chromatography (HPLC) needle (700 series, Hamilton CO., Reno, NV, USA). Contact angle measurements were taken immediately (within 30 s) after droplet dispensing. At least three different locations per sample were measured to assure consistency and reproducibility.

### 2.4. Lipid Printing and Rehydration

**Figure 1:**
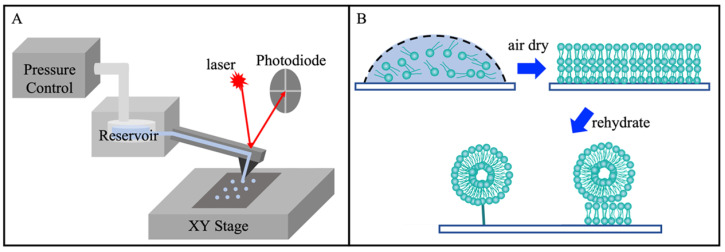
(**A**) Schematic diagram of an AFM combined with a microfluidic delivery. (**B**) Schematic diagram illustrating the key steps, following the delivery in (**A**), in forming liposomes via the rehydration of lipid nanostructures.

The lipid stock solutions were first prepared by dissolving POPC in chloroform to reach [POPC] = 3.3 × 10^−2^ M. NBD-PE stock solution was made by dissolving NBD-PE in chloroform to reach a concentration of 5.2 × 10^−3^ M. 39.5 µL of POPC and 2.5 µL NBD-PE stock solutions were mixed, then dried using nitrogen gas, yielding a yellow-colored POPC and NBD-PE solid mixture. Immediately before each delivery experiment, the mixture was dissolved with 40 µL of a mixed solvent, ethanol:glycerol = 9:1 (*v*:*v*), reaching a final concentration of [POPC] = 3.3 × 10^−2^ M, [NBD-PE] = 3.3 × 10^−4^ M to maintain dye:POPC = 1:100 (mole ratio). The delivery process was carried out by using a FluidFM BOT (Cytosurge, Glattbrugg, Switzerland), an atomic force microscopy (AFM)-based microfluidic delivery platform [44,45]. The key components included an inverted optical microscope (IX-73, Olympus America, Center Valley, PA, USA), a nanopipette with a 300 nm opening connected to the material reservoir, control units for an AFM probe contact and positioning, as well as material delivery (delivery pressure ranging from −800 mbar to 1000 mbar and contact time as short as 10 ms) [44,46,47,48]. Figure 1A illustrates the key components of the FluidFM BOT. The long-range XY-stage has a movable X range of 240 mm and a Y range of 74.5 mm, with precision down to 500 nm [38,44,49]. Rehydration was carried out by introducing an aqueous solution to the printed lipid constructs using a Microliter syringe (10 µL, 700 Series, Hamilton, Reno, NV, USA), as illustrated in Figure 1B. The rehydration solution was made by dissolving sucrose in a DPBS buffer at a final concentration of 500 mM. Sucrose is commonly used in the preparation of liposomes to maintain isosmotic conditions and due to its biocompatibility as well as its inertness toward lipid membranes [50].

### 2.5. Atomic Force Microscopy Imaging

The POPC constructs were left to air dry for 3–4 days and subsequently imaged on an AFM (MFP-3D, Oxford Instrument, Santa Barbara, CA, USA) for structural characterization. Microfabricated silicon nitride probes (MSNL-10 E, Bruker, Camarillo, CA, USA) were used to image the lipid structures. Images were acquired using the tapping mode with 56–78% damping. [38,40,51] Imaging processing, analysis, and display were carried out using the MFP-3D software developed on the Igor Pro 6.20 platform (WaveMetrics, Lake Oswego, OR, USA).

### 2.6. Laser Scanning Confocal Microscopy Imaging

A laser-scanning confocal microscope (LSCM) (FV-1000, Olympus America, Center Valley, PA, USA) was utilized to visualize the liposome rehydration process and products. Argon laser excitation at 458 nm and a 535–635 nm emission window were utilized to collect the NBD-PE fluorescent signal [52]. The images were taken under a 60X oil immersion objective at a 2 µs/pixel scanning speed, with 640 × 640 pixels in each image. Imaging processing, display and analysis were performed using the FV10-ASW Viewer software (Ver. 4.2b, Olympus America, Center Valley, PA, USA). A 3D display of liposomes was generated from the confocal images using Autodesk 3ds Max 2024 software (Autodesk, San Francisco, CA, USA).

## 3. Results and Discussion

### 3.1. Rehydration of Lipid Nanostructures Leads to Formation of Liposomes

**Figure 2:**
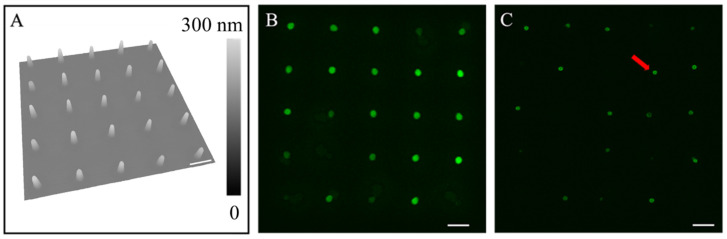
(**A**) A 3D display of an AFM topographic image of a 5 × 5 array of POPC constructs formed under a delivery pressure of −250 mbar. The AFM scan speed was set at 16.48 µm/s. (**B**) A 2D confocal scan of the POPC constructs array before rehydration, taken at z = 0.8 µm above the glass surface. (**C**) A 2D confocal scan of the liposome array 24 min after rehydration, taken at z = 0.8 µm above the glass surface. Scale bars = 5 µm.

Figure 2 shows an example of the successful production of tethered liposomes on plasma-cleaned glass surfaces by rehydrating engineered POPC nanoconstructs. A plasma-cleaned glass slide was used, whose contact angle measured as near zero for the POPC solutions. Delivery was carried out at delivery pressure *p* = −250 mbar and contact duration t = 150 ms. A 5 × 5 array containing 25 lipid constructs, with a periodicity of 10 µm, was first produced and then allowed to dry in clean ambient conditions for 3 days, after which AFM characterization was carried out. The frustum geometry was observed for individual lipid constructs, as shown in Figure 2A, from which the dimension of each construct was quantified. The diameters were measured (*n* = 25) to be 1.64 ± 0.05 µm at the bottom and 0.72 ± 0.08 µm at the top, and the height was measured to be 79.8 ± 9.8 nm. Rehydration was carried out 4 days after printing. After adding 5 µL of the rehydration solution to the side of the glass coverslip using a micro syringe, hydration occurred as the solution migrated to cover the entire surface. The rehydration process was monitored by time-lap LSCM, from which two images are shown in Figure 2B,C, representing before rehydration and 24 min after rehydration, respectively. For lipid constructs, d ≤ 3 μm, e.g., as shown in Figure 2, liposomes typically formed within 24 s upon contact with the rehydration solution, whose structure remained till the end of the experiment (30 min). Among the 25 nanoconstructs, 17 liposomes were clearly observed under LSCM 24 min after rehydration. The eight “missing” liposomes could be due to detachment during hydration or photobleaching. This event occurred for small constructs (lateral dimensions of less than 3 μm). In the cases of the “missing liposomes”, we searched the vicinity and could not locate them. The mechanical force experienced on these sites during rehydration might be responsible for the detachment and drafting away of these liposomes.

A representative liposome is indicated by a red arrow in Figure 2C, where the cross-sectional LSCM image clearly reveals the hollow center within. Our graphical abstract shows an example of a 3D view of these liposomes, i.e., definitive proof of the generation of liposomes. Among 25 lipid constructs, 17 liposomes remained on-site and had diameters measuring 0.81 ± 0.06 µm (*n* = 12, with the rest liposomes out of focus).

The results shown in Figure 2 demonstrate the successful production of liposomes by rehydrating engineered POPC nanoconstructs. A simple estimation comparing the numbers of POPC molecules in the constructs to those in the liposomes (SI) indicates that these liposomes are likely multilamellar. The experiments were repeated two times to assure reproducibility.

### 3.2. Liposomes Formed via Rehydrating Lipid Constructs Exhibit High Stability

**Figure 3:**
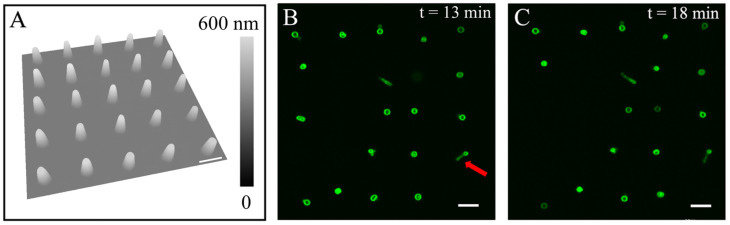
(**A**) A 3D display of an AFM topographic image of a 5 × 5 array of POPC constructs under a delivery pressure of 0 mbar. The AFM scan speed was set at 16.48 µm/s. (**B**) A 2D confocal scan of the liposome array 13 min after rehydration, taken at z = 0.8 µm above the glass surface. (**C**) A 2D confocal scan of the liposome array 18 min after rehydration, taken at z = 0.8 µm above the glass surface. Scale bars = 5 µm.

Using the same POPC solution formulation and under the same contact time as in Figure 2A, we delivered another 5 × 5 array (10 µm periodicity) of larger lipid constructs by increasing the extrusion pressure from *p* = −250 mbar to *p* = 0 mbar, as shown in Figure 3. All 25 lipid constructs exhibited a frustum geometry, as shown in Figure 3A. Among the 25 constructs, there were 19 hydrated features clearly visualized under LSCM, which remained in situ for the duration of the experiment (19 min after rehydration), as shown in Figure 3B (13 min) and 3C (18 min). The LSCM also indicates that most of liposomes remained at the initial delivery sites. Among the rest of the liposomes, the “tether string” was captured when the terminal liposomes moved away from their initial positions. In one example, as indicated by a red arrow in Figure 3B, the tether measured 2.2–2.9 µm in length, with liposome 0.8 µm above the surface and off-center by 1.73 µm (to the right of the center), as determined from the slice-by-slice LSCM imaging. These “tether strings” are likely elongated tubular structures formed via the self-organization of the POPC molecules. Similar observations were reported previously due to directional shear flow during rehydration [53,54,55]. Thus, we infer that our observed tether strings are likely lipo-tubes triggered by local and transient shear flow during the initial rehydration process.

The dimensions of the POPC constructs (*n* = 25) were quantified individually from the AFM topograph shown in Figure 3A; on average, the dimensions were 2.72 ± 0.11 µm for the bottom diameter, 1.25 ± 0.11 µm for the top diameter, and 215.3 ± 14.7 nm tall, respectively, i.e., doubling the volume of the frustums shown in Figure 2A. Upon rehydration, the diameter of the liposomes produced increased to d = 1.47 ± 0.12 µm (*n* = 9) (Figure 3B), representing a 72% increase as compared to the liposomes shown in Figure 2C. The comparison of the two series indicates that higher delivery pressure yields large POPC constructs and, in turn, bigger liposomes. Thus, these observations demonstrate the robustness of using our approach to produce liposomes. These tethered liposomes remained stable throughout the duration of the experiments, 18 min after hydration in the experiment shown in Figure 3 and 63 min after hydration in another test.

### 3.3. The Dimension of the Lipid Constructs Dictates the Liposomal Size

**Figure 4:**
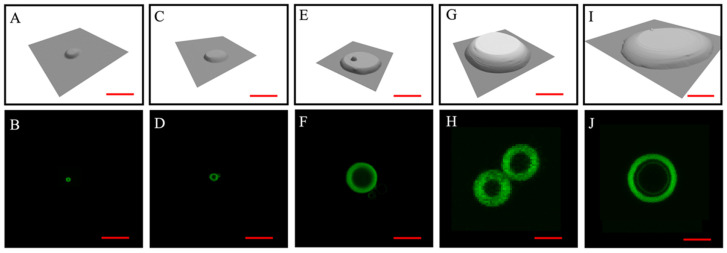
Top row, (**A**,**C**,**E**,**G**,**I**) are 3D displays of AFM topographs of five POPC constructs selected from the five representative experiments. The AFM scan speeds were set at 16.48, 23.96, 23.96, 16.00, and 24.00 µm/s, respectively, from left to right. Bottom row, (**B**,**D**,**F**,**H**,**J**) are the LSCM images of the liposomes formed by rehydrating the POPC construct shown in the image above. The LSCM scans were taken at the central plane of the vertical dimensions of each individual liposome. Scale bars = 5 µm.

A total of 84 sets of experiments were performed with various designs of POPC constructs, with lateral dimensions and heights of up to 1.5–20 µm and 80–900 nm, respectively. An array of nine (3 × 3) identical designs were produced per experiment among the first 11 sets and 25 (5 × 5) features per experiment for the remaining 73 sets. The POPC constructs were characterized using AFM topographic imaging. Rehydration was monitored by LSCM. Results from five experiments were selected and are shown in Figure 4 to represent five sizes within the range. Given the consistency observed among the individual features within the arrays (e.g., in Figure 2 and Figure 3) and for clarity, each frame in Figure 4 displays one feature from the corresponding array. For all the experiments, the same solution formulation and rehydration protocol as those in 3.1 were used.

The leftmost column of Figure 4 shows a tethered liposome (Figure 4B) with a liposomal diameter of 0.82 µm, rehydrated from a POPC construct (Figure 4A) measuring 1.73 µm for the bottom diameter, 0.74 µm for the top diameter and 76.0 nm for the height. This set of lipid constructs (a 5 × 5 array) was produced under *p* = −250 mbar and t = 150 ms on a plasma-cleaned surface. Constructs in the array, measuring 1.64 ± 0.05 µm for the bottom diameter, 0.72 ± 0.08 µm for the top diameter, and 79.8 ± 9.8 nm for the height, were rehydrated to form liposomes whose diameters measured 0.81 ± 0.06 µm each (*n* = 17). As the size of the lipid construct (Figure 4C) increased to 2.47 µm for the bottom diameter, 1.49 µm for the top diameter, and 202.9 nm for the height, a larger tethered liposome, whose diameter measured 1.45 µm (Figure 4D), was produced. This set of POPC constructs (a 5 × 5 array), generated under *p* = 100 mbar and t = 100 ms on a plasma-cleaned glass surface, measured 2.58 ± 0.12 µm for the bottom diameter, 1.28 ± 0.07 µm for the top diameter, and 159.9 ± 38.4 nm for the height. The rehydration of these constructs led to tethered liposomes whose diameters measured 1.42 ± 0.12 µm each (*n* = 9). Continuously increasing the dimensions of the POPC constructs to reach a bottom diameter = 6.52 µm, a top diameter = 5.12 µm, and a height = 161.2 nm (Figure 4E) led to even larger tethered liposomes, with dimension = 4.67 µm (Figure 4F). This set of POPC constructs (a 5 × 5 array), generated under *p* = 300 mbar and t = 100 mbar on a plasma-cleaned glass surface, measured 6.48 ± 0.28 µm for the bottom diameter, 4.91 ± 0.17 µm for the top diameter, and 172.0 ± 15.9 nm for the height. Rehydration of these constructs led to tethered liposomes with dimension = 4.67 ± 0.74 µm (*n* = 6). As shown in Figure 4G, as the size of the POPC construct further increased, the lipid construct measured 13.8 µm for the bottom diameter, 8.62 µm for the top diameter, and 847.2 nm for the height. Rehydration yields tethered double liposomes, both measuring 6.67 µm in diameter (Figure 4H). The array of 3 × 3 POPC constructs was produced under *p* = 800 mbar and t = 1000 ms on a plasma-cleaned glass surface. Four out of nine POPC constructs, each measuring 14.14 ± 0.34 µm for the bottom diameter, 8.87 ± 0.37 µm for the top diameter, and 886.7 ± 12.5 nm for the height, formed double liposomes in which the individual liposome diameters measured 6.21 ± 1.07 µm (*n* = 8) each. Finally, Figure 4J shows a single liposome from the same array as Figure 4G, with a liposomal diameter of 9.40 µm, formed via the rehydration of a lipid construct measuring 19.9 µm for the bottom diameter, 12.3 µm for the top diameter, and 443.9 nm for the height (Figure 4I). Three out of nine POPC constructs, each measuring 19.42 ± 0.49 µm for the bottom diameter, 14.15 ± 1.64 µm for the top diameter and 453.2 ± 52.3 nm for the height, formed single liposomes, in which each diameter measured 9.56 ± 0.23 µm (*n* = 3). For lipid constructs with lateral dimensions of 10–20 μm, e.g., in Figure 4H,J, the swelling time was 10–34 min, after which time the liposomal confocal images became steady. A simple estimation comparing the number of POPC molecules in constructs to those in the liposomes indicates multilamellarity among them.

In other experiments with the same lipid solution formulation, under *p* = 100 mbar and t = 100 ms, lipid constructs of 2.58 ± 0.12 µm in lateral dimension and 159.9 ± 38.4 nm in height were produced. The rehydration of such lipid constructs led to larger tethered liposomes, each of which had a diameter measuring 1.42 ± 0.12 µm (*n* = 9, Figure 4C,D). The rehydration of the lipid disks of the same design resulted in liposomes with almost the same diameters, in contrast to conventional methods which yielded wide distribution of liposomal size. The narrow size distribution suggests that the dimensions of the POPC constructs dictate the size of the liposomes formed via rehydration. Therefore, this new approach provides a reliable means to achieve size control in liposomal productions.

**Figure 5:**
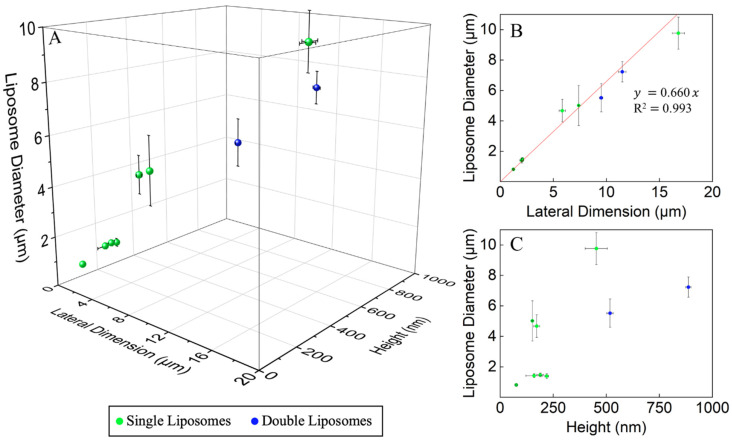
(**A**) Plotting of the liposome diameter against the lateral dimension and height of the POPC constructs. (**B**) Correlation between the liposome diameter and the lateral dimension of the POPC constructs, overlayed with the outcome of the linear regression. (**C**) Correlation between the liposome diameter and the height of the POPC constructs. The 3D plot shown in (**A**) was prepared using OriginPro 2025.

The correlation between the dimension of the liposomes and the POPC constructs is plotted in Figure 5A. We used the average of the base and top diameters to represent the lateral dimensions. With the increase in the base lateral dimension of the lipid construct, a clear and near-linear correlation is observed with the diameter of the liposome produced. Dissecting the impact of lateral dimension and height of POPC constructs, Figure 5B,C displays the lateral dimension dependence, and height dependence separately. For thin POPC constructs, the lateral dimensions show clear and near-linear correlation with the liposomal diameters. When the thickness of the POPC cakes reached 0.5 µm or thicker, double or triple liposomes were frequently observed. With the clear trend and quantitative correlation established, one could produce desired liposomes by design, i.e., by pre-engineering the lipid constructs and then rehydrating before the use of liposomes.

### 3.4. Potential to Produce Hierarchical Structures of Liposomes

**Figure 6:**
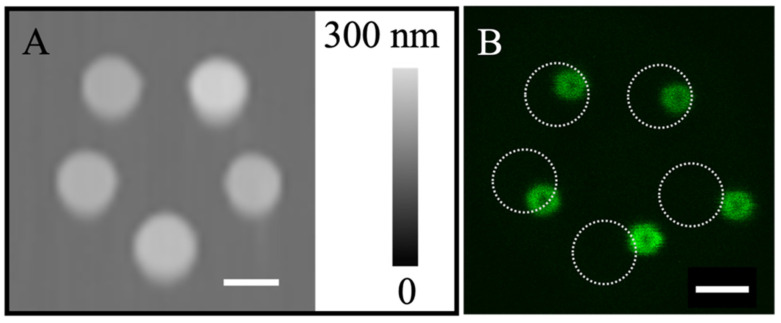
(**A**) AFM topographic image of five POPC constructs positioned at the vertices of a pentagon designed with side lengths of 2.0 µm. (**B**) Confocal scan of the liposome array 1 min after rehydrating the POPC constructs in (**A**), taken at z = 0.5 µm above the glass surface. The dotted circles indicate the original locations of each POPC construct. Scale bars = 1 µm.

Given the success and stability of the liposomes produced using our approach, we attempted to construct liposome hierarchical structures by arranging lipid constructs on pre-designed locations on surfaces. As shown in Figure 6A, the POPC constructs were positioned in the five corners of a pentagon, designed with side lengths of 2.0 µm. These constructs exhibited frustum geometry and measured 1.60 ± 0.05 µm in diameter and 72.3 ± 3.6 nm in height. The nearest neighbor separation among these POPC constructs measured 2.01 ± 0.16 µm, showing high fidelity to the design. The delivery pressure and time were *p* = −250 mbar and t = 150 ms, respectively. The same liquid formulation as shown in Figure 2 was used. Upon rehydration, five liposomes formed, as clearly visualized via LSCM and shown in Figure 6B. The liposomal diameter measured 0.80 ± 0.06 µm (*n* = 5). The liposome positions mostly followed the pentagon design (Figure 6B); the original position of the POPC constructs (indicated by the white dotted circles) are shown as a reference. The liposomes slightly shifted to the right upon rehydration. The nearest neighbor separation of the liposomes measured 1.73, 2.13, 1.76, 1.76, and 1.88 µm, respectively, starting from the top liposome and going clockwise. The deviations from the design and the locations of the POPC construct are likely due to the movement of these tethered liposomes from their original construct positions, following the flow of liquid during rehydration. The liposomes formed in this way were stable for at least 6 min (duration of the experiment). The robustness of our protocol was demonstrated by producing liposomes positioned at the vertices of pentagons and triangles (side length = 13.0 µm). Upon rehydration, liposomal pentagons and triangles were formed (Figure S5). The results collectively demonstrated the feasibility of using our approach to enable the construction of hierarchical liposomal structures by design.

## 4. Conclusions

The present work demonstrates the concept and feasibility of producing liposomes by rehydrating engineered POPC nanoconstructs. The shape and dimensions of the lipid constructs dictate the outcomes of the liposomes generated. Most of these liposomes produced remained attached or anchored to the surface, e.g., by a lipotether. The diameters of the liposomes exhibited near-linear correlations with the increase in the lateral dimension of the lipid constructs. Hierarchical liposomal structures can also be designed and produced by depositing lipid constructs to designated locations. Work is in progress to (a) correlate liposomal formation with other geometries and the intra-feature molecular packing of the lipid constructs, (b) expand the size range of liposomes produced and to determine the mechanism for the formation of single versus double liposomes, (c) control the detachment and collection of liposomes, and (d) produce liposomes inlaid with protein, ligands, and other composites. This new means of producing liposomes enables the achievement of designed sizes and composition, which shall advance the applications of drug delivery and liposome-based biomaterial engineering. The ability to produce hierarchical liposomal structures also paves the way for numerous applications such as proto-cell development, multiplexed bio-composite materials, and engineered local bio-environments.

## Data Availability

The original contributions presented in the study are included in the article, further inquiries can be directed to the corresponding author.

## Supplementary Materials

The following supporting information can be downloaded at: https://www.mdpi.com/article/10.3390/mi16020138/s1, Figure S1: Bright Field Images Corresponding to the LSCM Images Presented in Figure 3; Figure S2: Bright Field Images Corresponding to the LSCM Images Presented in Figure 4; Figure S3: Bright Field Images Corresponding to the LSCM Images Presented in Figure 6; Figure S4: LSCM Images of Complete POPC Construct Arrays Corresponding to Individual POPC Constructs Shown in Figure 4; Figure S5: LSCM Images of POPC Pentagon and Triangle Arrays Before and After Rehydration; Table S1: Calculated Results for The Number of Lipid Molecules in Lipid Frustums and Unilamellar Liposomes.
