# Supplementary material for: A New Means to Generate Liposomes by Rehydrating Engineered Lipid Nanoconstructs
Source: Micromachines (Basel). 2025 Jan 25;16(2):138. doi: 10.3390/mi16020138 (PMC11857817; doi:10.3390/mi16020138)
Supplement: Supplementary file 1 [file micromachines-16-00138-s001.zip › micromachines-3270579-supplementary.pdf]

Supplementary Information

A New Means to Generate Liposomes by Rehydrating Engineered Lipid Nanoconstructs

Yuqi Huang [1], Ziqian Xu [1], Umit Celik [1], Christopher F. Carnahan [2], Roland Faller [3,4], Atul N. Parikh [2,5] and Gang-yu Liu [1,2,*]

[1] Department of Chemistry, University of California, Davis, CA 95616, USA
[2] Biophysics Graduate Group, University of California, Davis, CA 95616, USA
[3] Department of Chemical Engineering, University of California, Davis, CA 95616, USA
[4] Department of Chemical Engineering, Texas Tech University, Lubbock, TX 79409, USA
[5] Department of Biomedical Engineering, University of California, Davis, CA 95616, USA
* Correspondence: gyliu@ucdavis.edu; Tel.: +1-(530)-754-9678

1. Liposome Lamellarity

The lamellarity of the liposomes produce can be determined through calculation based on AFM and confocal imaging data. For example, in Figure 4A, the POPC construct exhibits a frustum geometry with a bottom diameter $d_1 = 1.73 \pm 0.06$ μm, a top diameter $d_2 = 0.74 \pm 0.05$ μm, and a height $h = 76.0 \pm 1.3$ nm. (Note: Tip convolution was not taken into consideration due to the size of the frustum, hence the uncertainty is an underestimation.) The volume of an individual lipid frustum (V_f) can be calculated using Equation 1.

$$V_f = \frac{\pi h}{3}\left[\left(\frac{d_1}{2}\right)^2 + \left(\frac{d_2}{2}\right)^2 + \left(\frac{d_1}{2}\right)\left(\frac{d_2}{2}\right)\right] \tag{1}$$

The headgroup area of phosphatidylcholine ($a = 0.71$ nm^2) and the bilayer height ($h_b = 5$ nm) are used to estimate the number of POPC molecules in a single lipid frustum (N_f) according to Equation 2.

$$N_f = V_f\left(a \cdot \frac{h_b}{2}\right)^{-1} \tag{2}$$

Rehydration of the lipid frustum in Figure 4A led to a liposome whose diameter (d_l) were 0.82 μm. Given that the uncertainty of the confocal microscope is 500 nm, the measurement for liposomal diameter would have an uncertainty of 707 nm, as determined by principles of uncertainty propagation. Hypothetically, if the observed liposome were unilamellar, the number of lipid molecules it contains (N_u) could be calculated based on Equation 3.

$$N_u = \frac{4\pi}{a}\left[\left(\frac{d_l}{2}\right)^2 + \left(\frac{d_l}{2} - h_b\right)^2\right] \tag{3}$$

The calculation results corresponding to the structures in Figure 4A and 4B are shown in Table 1. Additonally, calculations were performed based on Figure 4I and 4J, where the lipid construct measured 19.91 ± 0.26 μm in bottom diameter, 12.28 ± 0.37 μm in top diameter, 443.9 ± 2.4 nm in height, and the liposome meaured 9.40 μm in diameter. The results are also included in Table 1.

Table 1. Calculated results for the number of lipid molecules in lipid frustums and unilamellar liposomes.

Corresponding Figures	V_f (nm^3)	N_f	N_u
Figure 4A, 4B	$(9.59 \pm 0.62) \times 10^7$	$(5.40 \pm 0.35) \times 10^7$	$(0.6 \pm 1.0) \times 10^7$
Figure 4I, 4J	$(9.20 \pm 0.25) \times 10^{10}$	$(5.18 \pm 0.14) \times 10^{10}$	$(8.4 \pm 1.2) \times 10^8$

As presented in Table 1, the number of POPC molecules in a lipid frustum (N_f) is larger than that in a unilamellar liposome (N_u) of the observed size. This suggests that the liposomes produced via the proposed rehydration method are multilamellar.

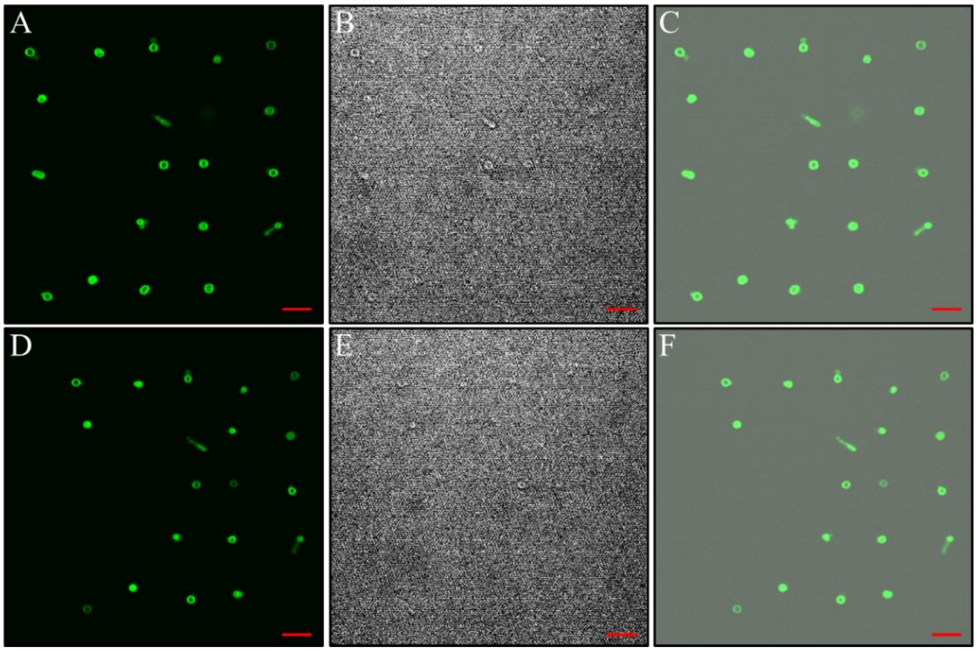

Figure S1. Left column, **(A)** and **(D)** are the LSCM images in Figure 3. Middle column, **(B)** and **(E)** are the bright field images corresponding to the LSCM images to the left. Right column, **(C)** and **(F)** are the overlayed LSCM and bright field images. Scale bars = 5 μm.

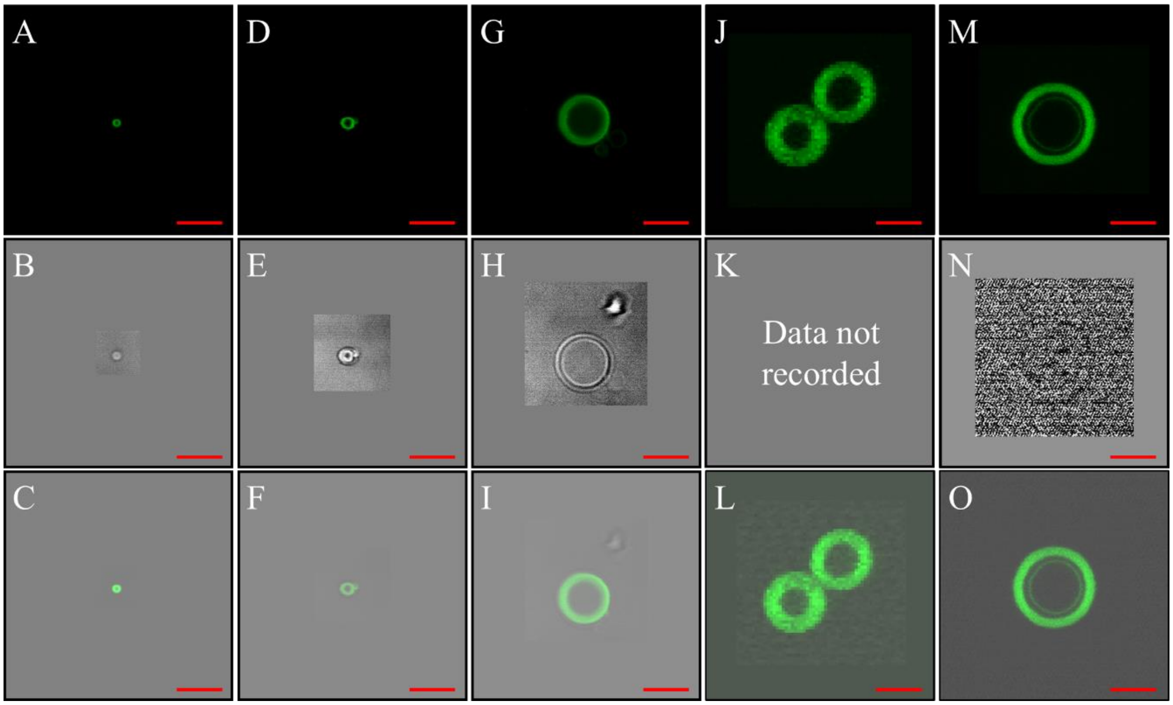

Figure S2. Top row, **(A), (D), (G), (J)** and **(M)** are the LSCM images in Figure 4. Middle row, **(B), (E), (H), (K)** and **(N)** are the bright field images corresponding to the LSCM images above. Bottom row, **(C), (F), (I), (L)** and **(O)** are the overlayed LSCM and bright field images. Scale bars = 5 μm.

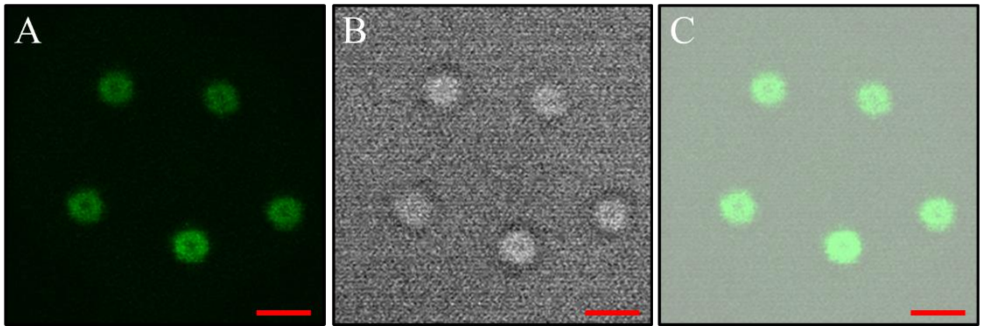

Figure S3. **(A)** The LSCM image in Figure 6. **(B)** The bright field images corresponding to (A). **(C)** The overlayed LSCM and bright field images. Scale bars = 1 μm.

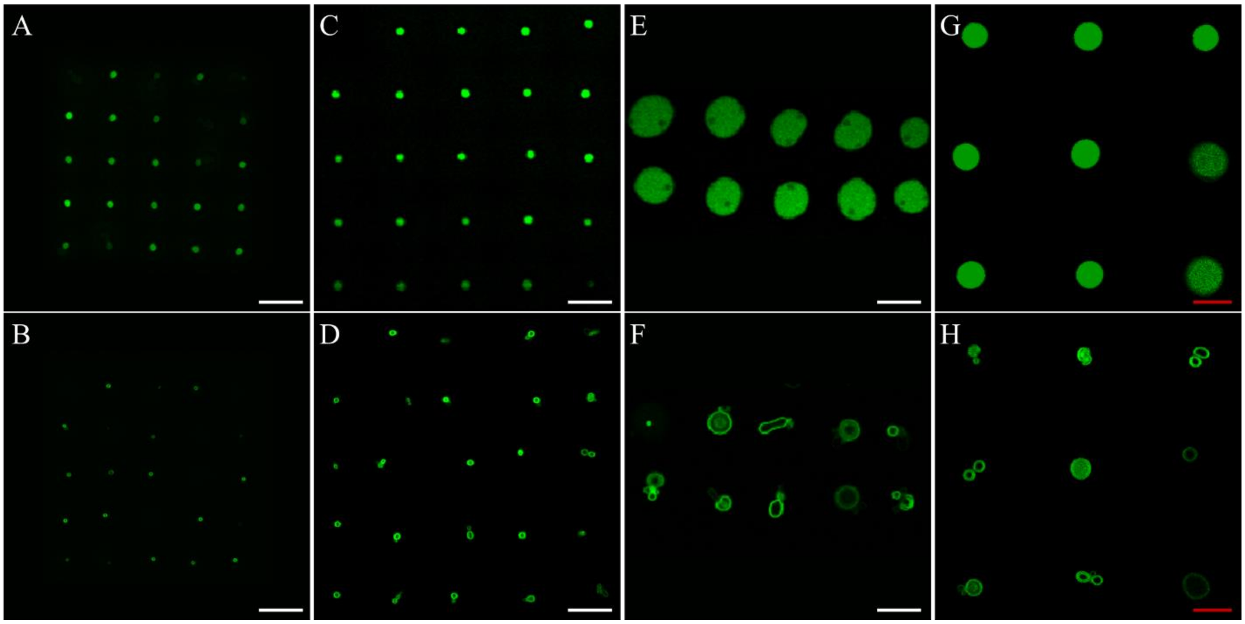

Figure S4. Top row, **(A)**, **(C)** and **(E)** are the LSCM images of POPC construct arrays corresponding to the single lipid constructs shown in Figure 4A, 4C and 4E, respectively. **(G)** is the LSCM image of POPC construct arrays corresponding to both Figure 4G and 4I. Bottom row, **(B), (D), (F)** and **(H)** are the LSCM images of the liposomes formed by rehydrating the POPC construct arrays shown in the image above. White scale bars = 10 μm. All images were acquired after liposomes reached steady strucures, except (F) where the confocal images was acquired at 3 min after hydration, thus the liposomes were still evolving. Red scale bars = 20 μm.

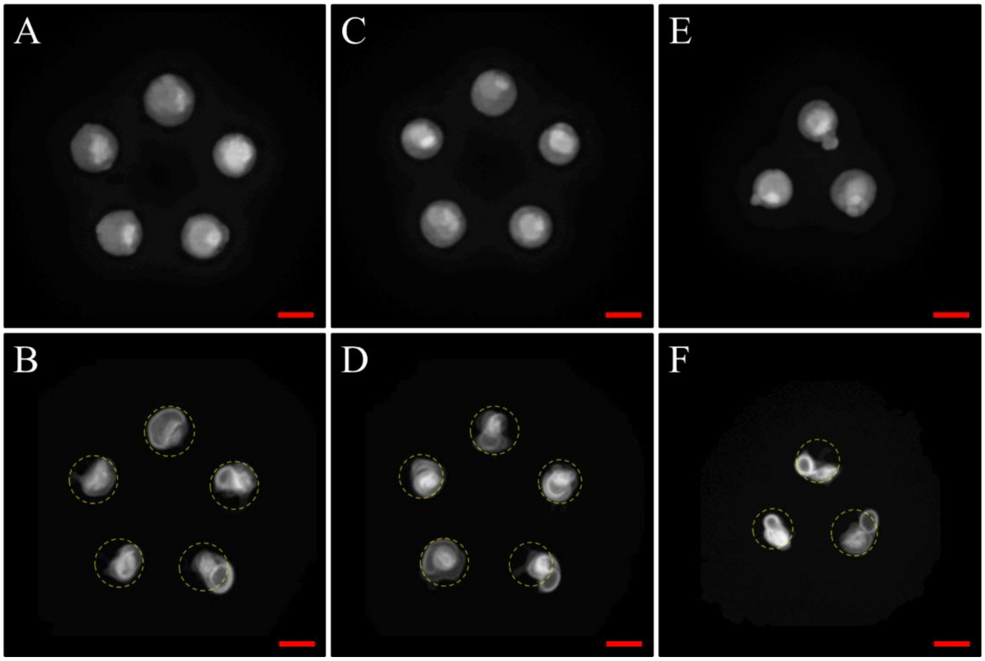

Figure S5. Top row, **(A)** and **(C)** are the LSCM images of two sets of five POPC constructs positioned at the vertices of a pentagon designed with side length of 13.0 μm. **(E)** is the LSCM image of three POPC constructs positioned at the vertices of a pentagon designed with side length of 13.0 μm. Bottom row, **(B),** **(D)** and **(H)** are the LSCM images of the liposome arrays formed by rehydrating the POPC construct arrays shown in the image above, acquired 20 min after hydration. The liposomes are still evolving as it would take 30-40 min to reach steady state. Scale bars = 5 μm.
